# Clinical profiling and outcomes of viral myocarditis manifesting with ventricular arrhythmias

**DOI:** 10.1093/ehjopen/oead132

**Published:** 2023-12-05

**Authors:** Giovanni Peretto, Simone Sala, Elisa Carturan, Stefania Rizzo, Andrea Villatore, Giacomo De Luca, Corrado Campochiaro, Anna Palmisano, Davide Vignale, Monica De Gaspari, Lorenzo Dagna, Antonio Esposito, Cristina Basso, Paolo Guido Camici, Paolo Della Bella

**Affiliations:** Department of Cardiac Electrophysiology and Arrhythmology, IRCCS San Raffaele Scientific Institute, Via Olgettina 60, 20132 Milan, Italy; Myocarditis Disease Unit, IRCCS San Raffaele Scientific Institute, Via Olgettina 60, 20132 Milan, Italy; School of Medicine, Vita-Salute San Raffaele University, Via Olgettina 60, 20132 Milan, Italy; Department of Cardiac Electrophysiology and Arrhythmology, IRCCS San Raffaele Scientific Institute, Via Olgettina 60, 20132 Milan, Italy; Myocarditis Disease Unit, IRCCS San Raffaele Scientific Institute, Via Olgettina 60, 20132 Milan, Italy; Cardiovascular Pathology, Department of Cardio-Thoracic-Vascular Sciences & Public Health and Azienda Ospedaliera, University of Padua Medical School, Padua, Italy; Cardiovascular Pathology, Department of Cardio-Thoracic-Vascular Sciences & Public Health and Azienda Ospedaliera, University of Padua Medical School, Padua, Italy; Myocarditis Disease Unit, IRCCS San Raffaele Scientific Institute, Via Olgettina 60, 20132 Milan, Italy; School of Medicine, Vita-Salute San Raffaele University, Via Olgettina 60, 20132 Milan, Italy; Myocarditis Disease Unit, IRCCS San Raffaele Scientific Institute, Via Olgettina 60, 20132 Milan, Italy; School of Medicine, Vita-Salute San Raffaele University, Via Olgettina 60, 20132 Milan, Italy; Unit of Immunology, Rheumatology, Allergy and Rare Diseases (UnIRAR), IRCCS San Raffaele Scientific Institute, Milan, Italy; Myocarditis Disease Unit, IRCCS San Raffaele Scientific Institute, Via Olgettina 60, 20132 Milan, Italy; School of Medicine, Vita-Salute San Raffaele University, Via Olgettina 60, 20132 Milan, Italy; Unit of Immunology, Rheumatology, Allergy and Rare Diseases (UnIRAR), IRCCS San Raffaele Scientific Institute, Milan, Italy; Myocarditis Disease Unit, IRCCS San Raffaele Scientific Institute, Via Olgettina 60, 20132 Milan, Italy; School of Medicine, Vita-Salute San Raffaele University, Via Olgettina 60, 20132 Milan, Italy; Experimental Imaging Center, Radiology Unit, IRCCS San Raffaele Scientific Institute, Milan, Italy; Myocarditis Disease Unit, IRCCS San Raffaele Scientific Institute, Via Olgettina 60, 20132 Milan, Italy; Experimental Imaging Center, Radiology Unit, IRCCS San Raffaele Scientific Institute, Milan, Italy; Cardiovascular Pathology, Department of Cardio-Thoracic-Vascular Sciences & Public Health and Azienda Ospedaliera, University of Padua Medical School, Padua, Italy; School of Medicine, Vita-Salute San Raffaele University, Via Olgettina 60, 20132 Milan, Italy; Unit of Immunology, Rheumatology, Allergy and Rare Diseases (UnIRAR), IRCCS San Raffaele Scientific Institute, Milan, Italy; Myocarditis Disease Unit, IRCCS San Raffaele Scientific Institute, Via Olgettina 60, 20132 Milan, Italy; School of Medicine, Vita-Salute San Raffaele University, Via Olgettina 60, 20132 Milan, Italy; Experimental Imaging Center, Radiology Unit, IRCCS San Raffaele Scientific Institute, Milan, Italy; Cardiovascular Pathology, Department of Cardio-Thoracic-Vascular Sciences & Public Health and Azienda Ospedaliera, University of Padua Medical School, Padua, Italy; Cardiovascular Research Center, IRCCS San Raffaele Scientific Institute, Milan, Italy; Department of Cardiac Electrophysiology and Arrhythmology, IRCCS San Raffaele Scientific Institute, Via Olgettina 60, 20132 Milan, Italy

**Keywords:** Myocarditis, Viral, Ventricular arrhythmias, Sudden cardiac death, Endomyocardial biopsy

## Abstract

**Aims:**

Clinical features and risk stratification of patients with viral myocarditis (VM) complicated by ventricular arrhythmias (VA) are incompletely understood. We aim to describe arrhythmia patterns and outcomes in patients with VM and early-onset VA.

**Methods and results:**

We present a single-centre study, enrolling patients with VM proven by endomyocardial biopsy, and evidence of VA within 24 h of hospitalization. The incidence of major adverse events (MAE), including all-cause death, severe heart failure, advanced atrioventricular blocks, or major VA, was evaluated during a 24-month follow-up (FU) and compared with a matched group of virus-negative myocarditis. Of patients with VM (*n* = 74, mean age 47 ± 16 years, 66% males, and left ventricular ejection fraction 51 ± 13%), 20 (27%) presented with major VA [ventricular tachycardia/ventricular fibrillation (VT/VF)], and 32 (44%) had polymorphic VA. Patients with polymorphic VA more commonly had evidence of ongoing systemic infection (24/32 vs. 10/42, *P* = 0.004) and experienced greater occurrence of MAE at discharge (15/32 vs. 2/42, *P* < 0.001). However, the incidence of MAE during FU was higher in patients with monomorphic VA compared to those with polymorphic VA (17/42 vs. 2/28, *P* = 0.002). Patients with monomorphic VA displayed frequently signs of chronic cardiomyopathy and had outcomes comparable with virus-negative myocarditis (log rank *P* = 0.929). Presentation with VT/VF was independently associated with MAE [at discharge: hazard ratio (HR) 4.7, 95% confidence interval (CI) 1.6–14.0, *P* = 0.005; during FU: HR 6.3, 95% CI 2.3–17.6, *P* < 0.001].

**Conclusion:**

In patients with VM, polymorphic VA point to ongoing systemic infection and early adverse outcomes, whereas monomorphic VA suggest chronic cardiomyopathy and greater incidence of MAE during FU. Presentation with VT/VF is independently associated with MAE.

## Graphical Abstract

**Graphical Abstract:**
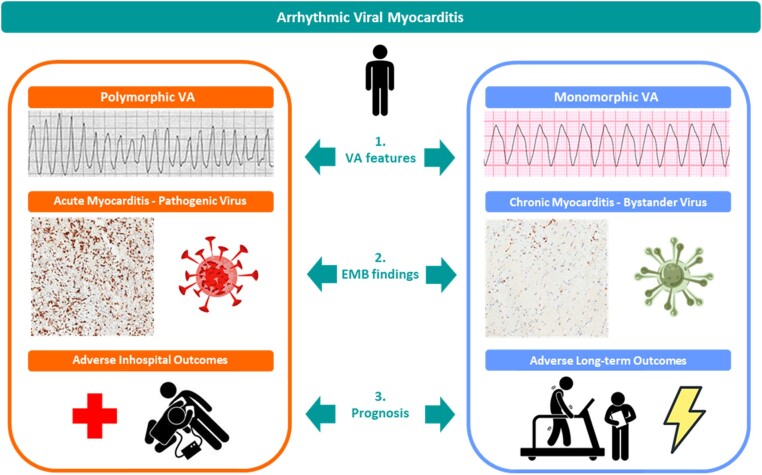

## Introduction

Ventricular arrhythmias (VA) may complicate the clinical course of myocarditis, resulting in an increased risk of mortality and morbidity.^1,2^ Recently, it has been shown that distinct electrocardiographic features of VA at the onset of myocarditis are in close relationship with myocardial inflammatory status.^3^ In particular, while polymorphic VA are dominant during active myocarditis, monomorphic VA are more common in the post-inflammatory stage of the disease.^3^ As a consequence, the application of patient-tailored treatment strategies, ranging from immunosuppression to catheter ablation of VA, has been shown to significantly influence outcomes.^4,5^

The above-defined strategies mainly apply to biopsy-proven virus-negative or autoimmune myocarditis^2–5^ whereas the arrhythmic manifestations of viral myocarditis (VM) have not been systematically investigated.^6^ The issue is relevant, since viruses constitute a common aetiology of myocarditis and play a relevant prognostic role.^6,7^ In fact, viral infections have been frequently reported in complicated presentations of myocarditis.^8^ In addition, the persistence of viral genomes within the myocardium has been depicted as a major driving force for the evolution towards dilated cardiomyopathy and heart failure late after presentation.^9^ Yet, no data are currently available about the short- and long-term occurrence of arrhythmic events in patients with VM.^6^

The aims of the present study are (i) to characterize VA complicating the early onset of VM, and analyse their relationships with distinct viral aetiologies, and (ii) to report both short- and long-term outcomes of the arrhythmic variant of VM.

## Methods

### Study description

**Figure 1:**
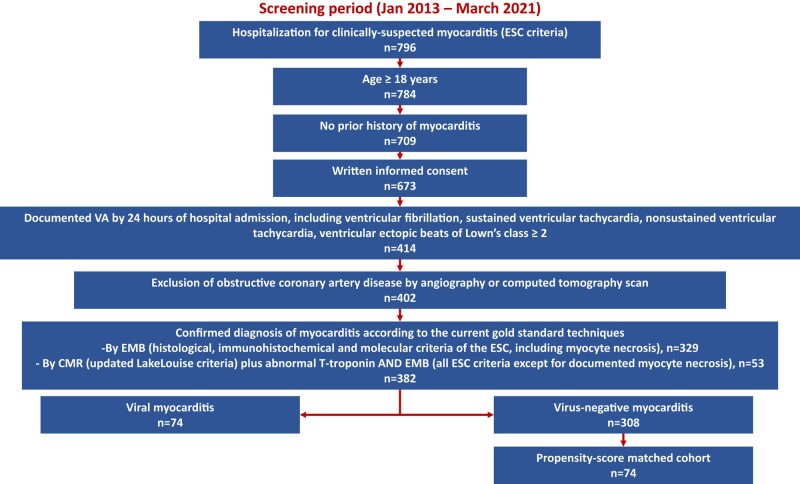
Study flowchart. The study flowchart is shown. Further details about diagnostic criteria are reported in the Supplementary material. CMR, cardiac magnetic resonance; EMB, endomyocardial biopsy; ESC, European Society of Cardiology; VA, ventricular arrhythmias.

We present a single-centre, prospective study, taking place at a tertiary referral centre for VA management, and with dedicated multidisciplinary facilities for the diagnosis and treatment of myocarditis.^10^ This study complies with the Declaration of Helsinki and was approved by the local institutional review board. From January 2013 to March 2021, we screened consecutive inpatients with clinically suspected myocarditis based on the 2013 European Society of Cardiology (ESC) criteria.^2^ The study flowchart is shown in *Figure 1*. The inclusion criteria for enrollment were as follows: (i) age ≥ 18 years; (ii) no prior history of myocarditis; (iii) written informed consent; (iv) documentation of VA within 24 h of hospital admission, including any of the following: ventricular fibrillation (VF), sustained ventricular tachycardia (VT), non-sustained VT (NSVT), and ventricular ectopic beats (VEBs) of Lown’s class ≥ 2 (i.e. >1 VEB/min or >30 VEBs/h)^11^; (v) exclusion of obstructive coronary artery disease, either by angiography or computed tomography; and (vi) confirmed diagnosis of myocarditis by gold standard techniques (detail below). Patients with VM constituted the study group. A propensity score–matched cohort of patients with virus-negative myocarditis served as comparator group.

### Diagnosis of myocarditis

All patients underwent both endomyocardial biopsy (EMB) and cardiac magnetic resonance (CMR) on top of routine diagnostic workup. For the study purposes, EMB was considered as the reference technique for diagnosing myocarditis, according to the gold standard histological (Dallas criteria), immunohistochemical (≥14 leucocytes/mm^2^ and CD3^+^ T lymphocytes ≥ 7 cells/mm^2^), and molecular criteria of the ESC.^2^ Whenever the lack of myocyte necrosis on EMB was deemed due to sampling errors,^12^ the updated Lake Louise criteria^13^ on CMR in association with abnormal values of T-troponin were used to confirm the diagnosis of clinically suspected myocarditis. In compliance with the current diagnostic recommendations for VM,^2,6^ a uniform panel of viral genomes was screened by polymerase chain reaction in both EMB tissue and blood samples. Further details about diagnostic workup and classification of viruses are reported in the Supplementary material.

### Characterization of ventricular arrhythmias

As per local standard practice, all patients underwent continuous 12-lead electrocardiogram (ECG) telemonitoring for the detection of arrhythmias during hospital stay. As previously reported,^3^ VA were classified into monomorphic, in the presence of a single 12-lead ECG morphology for at least 75% of the recorded beats; polymorphic otherwise. The classification is consistent with updated definitions.^14^ In the event of discordance between multiple VA types, the analysis of morphology was performed on the clinically dominant arrhythmia (i.e. VT/VF > NSVT > VEBs). Electrocardiogram recordings were independently analysed by two electrophysiologists blinded to the study design and outcomes. A third physician was involved in case of discordancy.

### Treatment and follow-up

On top of ESC guideline-based optimal medical treatment for heart failure and arrhythmias,^1,15^ aetiology-driven treatment choices were evaluated, case by case, by a specialized multidisciplinary ‘disease unit’ for myocarditis.^10^ Aetiology-driven treatment options for VM were discussed with virologists and included specific antiviral agents, intravenous immunoglobulins (IVIG), or immunomodulatory therapies (IMT).^2,16^ For the comparator cohort of virus-negative myocarditis, classic immunosuppressive drug regimens were used, including prednisone, azathioprine, and mycophenolate mofetil.^17^ Invasive therapeutic strategies, such as implant of cardiac devices and catheter ablation of arrhythmias, were in compliance with the updated recommendations.^1,2,14^ All patients were prospectively evaluated every 3 months while on aetiology-driven therapy and every 6 months otherwise for a total follow-up (FU) of 24 months. Multimodal re-assessment included blood exams, echocardiogram, 24-h Holter ECG, cardiac device telemonitoring whenever applicable, and either invasive or non-invasive restaging of myocarditis.

### Endpoints

The primary study endpoints included (i) the prevalence of polymorphic VA at baseline evaluation, in relation to specific VM aetiologies, and (ii) the occurrence of major adverse events (MAE), which were independently evaluated by discharge from hospital (after documentation of the first VA) and during FU by 24 months. Major adverse events included the following: (i) all-cause death; (ii) severe heart failure, requiring inotropic or mechanical circulatory support (before discharge) or causing re-hospitalization (during FU); (iii) advanced (i.e. second Mobitz II or third degree) atrioventricular blocks (AVB); and (iv) major VA, including VF, sustained VT, or appropriate antitachycardia pacing or shock in implantable cardioverter defibrillator (ICD) carriers.

### Statistical analysis

SPSS Version 20 (IBM Corp., Armonk, NY) was used for analysis. Continuous variables were expressed as mean or median with standard deviation (SD) or range, depending on the distribution of data, as assessed by the Shapiro–Wilk test. Accordingly, they were compared by parametric (Student’s *t*) or non-parametric (Mann–Whitney *U*) tests, respectively. Categorical variables are reported as counts and percentages and were compared by Fisher’s exact test. To identify 1:1 matched controls with virus-negative myocarditis, the propensity score method was used, accounting for the following baseline covariates: age, sex, clinical presentation, baseline left ventricular ejection fraction (LVEF), and VA type (further details in the Supplementary material and Supplementary material online, *Table S1*). Survival curves were generated by the Kaplan–Meier method and compared by the log rank test. To assess the 24-month occurrence of MAE, the FU time was calculated from the date of discharge from the hospital. Patients undergoing inhospital death or catheter ablation of VA were excluded from long-term FU analysis. The Cox proportional hazard model was used for multivariable analysis. The covariates of risk stratification were chosen based on the known clinical relevance (Supplementary material). Confidence intervals (CI) were set at 95%. Two-sided *P* < 0.05 was judged as statistically significant.

## Results

### Baseline clinical features

Out of 382 patients with confirmed diagnosis of myocarditis, 74 cases (19%) had VM (mean age 47 ± 16 years, 66% males). In particular, the diagnosis of myocarditis was proven by EMB in all cases (including 38% with missing necrosis) and also by CMR in 65/74 (88%). At baseline evaluation, the mean LVEF was 51 ± 13%, and 33 patients (45%) had presentation complicated either by acute heart failure, LVEF < 50%, or sustained VT/VF.^8^ In detail, documented VA by 24 h of hospital admission included VF (*n* = 6), sustained VT (*n* = 14), NSVT (*n* = 29), and frequent VEBs (*n* = 25). With respect of their clinically dominant arrhythmia, 32 patients (44%) had polymorphic VA. Given the coefficient of concordancy *k* = 1 between ECG analysts, the third judgement about VA morphology was never required. Full details about clinical presentation and diagnostic workup are shown in *Table 1*. The most common viral aetiologies included parvovirus B19 (57%) and herpesviruses (27%), with 10 patients (14%) showing multiple viral genomes. Further details are shown in Supplementary material online, *Figure S1*.

**Table 1:** Baseline features of patients with viral myocarditis and polymorphic vs. monomorphic ventricular arrhythmias

	P-VA (*n* = 32)	M-VA (*n* = 42)	*P*	Total VM (*n* = 74)	Comparator VNM (*n* = 74)	*P*
Clinical profile						
Age (years)	46 ± 16	47 ± 15	0.783	47 ± 16	47 ± 15	1.000
Male sex	19 (59)	30 (71)	0.326	49 (66)	48 (65)	1.000
Caucasian	27 (84)	39 (93)	0.280	66 (89)	68 (92)	0.780
Associated SR/HDs	4 (13)	5 (12)	1.000	9 (12)	11 (15)	0.811
History of SCD/CMP	1 (3)	3 (7)	1.000	4 (5)	3 (4)	1.000
Presentation						
Chest pain	12 (38)	15 (36)	1.000	27 (36)	26 (35)	1.000
Dyspnoea	12 (38)	9 (21)	0.193	21 (28)	21 (28)	1.000
Syncope/palpitation	8 (25)	18 (43)	0.143	26 (35)	27 (36)	1.000
Complicated^a^	16 (50)	17 (40)	0.483	33 (45)	33 (45)	1.000
Fever in the last 30 days	16 (50)	6 (14)	**0.002**	22 (30)	4 (5)	**<0**.**001**
VA documented by 24 h of admission						
VF	6 (19)	0 (0)	**0**.**005**	6 (8)	1 (1)	0.116
Sustained VT	5 (16)	9 (21)	0.566	14 (19)	20 (27)	0.329
NSVT	10 (31)	19 (45)	0.241	29 (39)	31 (42)	0.867
Lown’s grade ≥ 2^a^ VEBs	11 (43)	14 (33)	1.000	25 (34)	27 (36)	0.863
VEB daily burden (10^3^)	1.2 (0.4–3.1)	1.0 (0.3–3.6)	0.695	1.1 (0.3–3.4)	1.3 (0.4–3.8)	0.761
Baseline ECG and other arrhythmias						
PQ (ms)	173 ± 40	179 ± 38	0.513	175 ± 39	179 ± 41	0.544
QRS duration (ms)	102 ± 22	104 ± 21	0.913	103 ± 21	105 ± 23	0.582
QTc (ms)	416 ± 34	409 ± 32	0.425	411 ± 32	409 ± 33	0.709
LBBB	2 (6)	3 (7)	1.000	5 (7)	6 (8)	1.000
AF	1 (3)	2 (5)	1.000	3 (4)	4 (5)	1.000
Blood exams						
T-troponin (ng/L)	46 (16–314)	38 (9–255)	0.734	41 (13–286)	35 (10–268)	0.813
NT-proBNP (pg/mL)	429 (123–1812)	348 (100–1399)	0.624	399 (106–1545)	313 (101–1499)	0.792
C-reactive protein (mg/L)	12 (6–23)	5 (3–16)	**0**.**026**	7 (4–19)	5 (3–17)	0.566
ESR (mm/h)	13 (6–26)	11 (5–24)	0.711	12 (5–26)	10 (5–24)	0.723
Echocardiogram						
LVEDVi (mL/m^2^)	64 ± 22	67 ± 20	0.542	66 ± 21	68 ± 22	0.573
LVEF (%)	46 ± 14	55 ± 12	**0**.**004**	51 ± 13	51 ± 13	1.000
LVEF < 50%	12 (38)	10 (24)	0.305	22 (30)	22 (30)	1.000
E/E′	9 ± 4	8 ± 4	0.290	8 ± 4	8 ± 3	1.000
TAPSE (mm)	21 ± 4	22 ± 3	0.223	22 ± 4	22 ± 4	1.000
Pericardial effusion	3 (9)	6 (14)	0.723	9 (12)	11 (15)	0.811
Cardiac magnetic resonance						
Updated LLC+	31 (97)	34 (81)	0.069	65 (88)	59 (79)	0.265
T_2_-STIR+	23 (72)	23 (55)	0.154	46 (62)	40 (54)	0.405
LGE+	31 (97)	42 (100)	1.000	73 (99)	74 (100)	1.000
Septal LGE	13 (41)	19 (45)	0.814	32 (43)	34 (46)	0.869
T_2_ > 50 ms	18/20 (90)	20/24 (83)	0.673	38/44 (86)	33/41 (80)	0.564
Native T_1_ > 1045 ms	19/20 (95)	21/24 (88)	0.614	40/44 (91)	37/41 (90)	1.000
ECV > 27%	16/20 (80)	20/24 (83)	1.000	36/44 (82)	35/41 (85)	0.386
Endomyocardial biopsy						
Lymphocytic histotype	32 (100)	42 (100)	1.000	74 (100)	74 (100)	1.000
CD3^+^ > 7/mm^2^	32 (100)	42 (100)	1.000	74 (100)	74 (100)	1.000
Necrosis	24 (75)	22 (52)	0.056	46 (62)	38 (51)	0.245
Replacement fibrosis	8 (25)	30 (71)	**<0**.**001**	38 (51)	56 (72)	**0**.**004**
Myocyte hypertrophy	5 (16)	15 (36)	0.067	20 (27)	28 (38)	0.219
Viral genomes	32 (100)	42 (100)	1.000	74 (100)	0 (0)	**<0**.**001**

Comparison is shown between VM with P-VA vs. M-VA, as well as between VM and comparator group of VNM. Measures are mean ± standard deviation, median (quartile 1–quartile 3), or count (%). Significant differences are enhanced in bold font.

AF, atrial fibrillation; CD, cluster of differentiation; ECV, extracellular volume; ESR, erythrocyte sedimentation rate; LBBB, left bundle branch abnormality; LGE, late gadolinium enhancement; LLC, Lake Louise criteria; LVEDVi, left ventricular end-diastolic volume indexed; LVEF, left ventricular ejection fraction; M-VA, monomorphic ventricular arrhythmias; NSVT, non-sustained ventricular tachycardia; P-VA, polymorphic ventricular arrhythmias; SCD/CMP, sudden cardiac death/cardiomyopathy; SR/HDs, systemic rheumatologic/hematologic diseases; STIR, short-tau inversion recovery; TAPSE, tricuspid annular plane systolic excursion; VEBs, ventricular ectopic beats; VF, ventricular fibrillation; VM, viral myocarditis; VNM, virus-negative myocarditis; VT, ventricular tachycardia.

^a^Complicated presentation included either acute heart failure, LVEF < 50%, or sustained VT/VF.^8^

### Relationships between ventricular arrhythmia features and viral myocarditis aetiology

**Figure 2:**
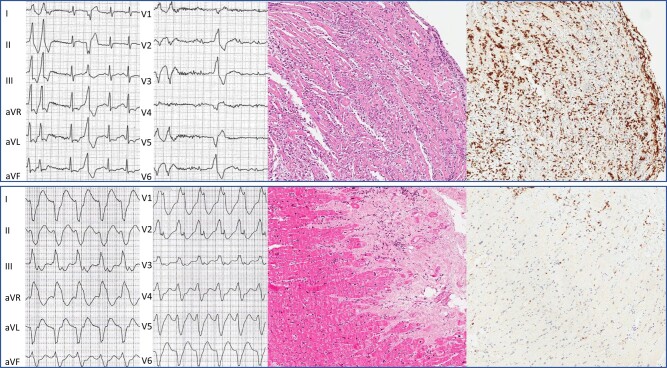
Representative examples of the spectrum of ventricular arrhythmias in viral myocarditis. Representative examples of ventricular arrhythmias in patients with different stages and viral aetiologies are shown. In particular, from the left to the right, the upper and lower panels show (i) 12-lead electrocardiogram features of arrhythmias; (ii) haematoxylin–eosin assay on endomyocardial biopsy; and (iii) immunohistochemistry assay on endomyocardial biopsy with CD3^+^ T lymphocytes labelled. Upper panel: polymorphic ventricular ectopic beats in a patient with fever and endomyocardial biopsy–proven acute myocarditis caused by adenovirus. Endomyocardial biopsy shows high prevalence of inflammatory infiltrates and trivial fibrosis. Lower panel: monomorphic ventricular tachycardia in a patient with endomyocardial biopsy–proven chronic myocarditis and evidence of low-load (i.e. <500 copies/µg) parvovirus B19. Endomyocardial biopsy shows high prevalence of replacement fibrosis and low degree of lymphocytic inflammatory infiltrates. This patient presented with no signs or symptoms of systemic infection. CD, cluster of differentiation.

The relationships between VA features and VM aetiology are shown in *Table 2*. As the most significant finding, patients with polymorphic VA more commonly manifested signs of ongoing systemic infection, including fever and higher levels of C-reactive protein, compared to cases showing monomorphic VA (24/32 vs. 10/42, respectively; *P* = 0.004; *Table 1*). In addition, patients with polymorphic VA had higher prevalence of cardiotropic viral genomes (mainly adenoviruses) compared to cases showing monomorphic VA (11/32 vs. 3/42, respectively; *P* = 0.006). Conversely, patients with monomorphic VA had higher prevalence of low-load (i.e. <500 copies/µg) parvovirus B19 genome (22/42 vs. 5/32; *P* = 0.001), replacement fibrosis (71% vs. 25%, *P* < 0.001), and additional findings on EMB suggesting chronic inflammatory cardiomyopathy, compared to patients with polymorphic VA (*Table 1*). Representative examples of the relationships between VA features and distinct viral aetiologies are shown in *Figure 2*.

**Table 2:** Relationships between baseline ventricular arrhythmias and viruses

	Total (*n* = 74)	P-VA (*n* = 32)	M-VA (*n* = 42)	*P*	VT/VF (*n* = 20)	NSVT/VEB (*n* = 54)	*P*
Systemic infection	34 (46)	24 (75)	10 (24)	**0.004**	11 (55)	23 (43)	0.433
Spring	21 (28)	13 (41)	8 (19)	0.067	6 (30)	15 (28)	1.000
Summer	10 (14)	3 (9)	7 (17)	0.499	1 (5)	9 (17)	0.270
Autumn	25 (34)	6 (19)	19 (45)	**0**.**025**	8 (40)	17 (31)	0.583
Winter	18 (24)	10 (31)	8 (19)	0.279	5 (25)	13 (24)	1.000
DNA viruses	64 (86)	28 (88)	36 (86)	1.000	17 (85)	47 (87)	1.000
RNA viruses	12 (26)	6 (19)	6 (14)	0.752	4 (20)	8 (15)	0.724
Cardiotropic viruses	14 (19)	11 (34)	3 (7)	**0**.**006**	5 (25)	9 (17)	0.506
Vasculotropic viruses	42 (57)	14 (44)	28 (67)	0.061	8 (40)	34 (63)	0.113
Lymphotropic viruses	19 (26)	10 (31)	9 (21)	0.082	9 (45)	10 (19)	**0**.**034**
Cardiotoxic viruses	9 (12)	6 (19)	3 (7)	0.163	3 (15)	6 (11)	0.696
Multiple viral genomes	10 (14)	8 (25)	2 (5)	**0**.**016**	4 (20)	6 (11)	0.444
Adenovirus	11 (15)	8 (25)	3 (7)	**0**.**048**	4 (20)	7 (13)	0.475
Cytomegalovirus	10 (14)	4 (13)	6 (14)	1.000	5 (25)	5 (9)	0.122
Enterovirus	3 (4)	3 (9)	0 (0)	0.077	1 (5)	2 (4)	1.000
Epstein–Barr virus	1 (1)	1 (3)	0 (0)	0.432	0 (0)	1 (2)	1.000
Hepatitis C virus	1 (1)	1 (3)	0 (0)	0.432	1 (5)	0 (0)	0.270
Herpes virus-1	2 (3)	1 (3)	1 (2)	1.000	0 (0)	2 (4)	1.000
Herpes virus-6, all	5 (7)	2 (6)	3 (7)	1.000	3 (15)	2 (4)	0.119
Herpes virus-6, low-load^a^	3 (4)	0 (0)	3 (7)	0.254	2 (10)	1 (2)	0.176
Herpes virus-6, high-load^a^	2 (3)	2 (6)	0 (0)	0.184	1 (5)	1 (2)	0.470
HIV	4 (5)	2 (6)	2 (5)	1.000	1 (5)	3 (6)	1.000
Influenza virus A	1 (1)	1 (3)	0 (0)	0.432	1 (5)	0 (0)	0.270
Parainfluenza virus	1 (1)	1 (3)	0 (0)	0.432	0 (0)	1 (2)	1.000
Parvovirus B19, all	42 (57)	14 (44)	28 (67)	0.061	8 (40)	34 (63)	0.113
Parvovirus B19, low-load^a^	27 (36)	5 (16)	22 (52)	**0**.**001**	5 (25)	22 (41)	0.281
Parvovirus B19, high-load^a^	15 (20)	9 (28)	6 (14)	0.158	3 (15)	12 (22)	0.746
SARS-Coronavirus-2	2 (3)	1 (3)	1 (2)	1.000	0 (0)	2 (4)	1.000
Varicella zoster virus	2 (3)	2 (6)	0 (0)	0.184	1 (5)	1 (2)	1.470

Relationships between baseline VA features and viral aetiologies are shown. Measures are count (%). Significant differences are enhanced in bold font.

DNA, deoxyribonucleic acid; HIV, human immunodeficiency virus; M-VA, monomorphic ventricular arrhythmias; NSVT/VEB, non-sustained ventricular tachycardia/ventricular ectopic beats; P-VA, polymorphic ventricular arrhythmias; RNA, ribonucleic acid; SARS, severe acute respiratory syndrome; VT/VF, ventricular tachycardia/ventricular fibrillation.

^a^Low-load: <500 copies/μg, high-load: >500 copies/μg.

### Treatment

Optimal medical treatment included renin–angiotensin–aldosterone inhibitors, beta-blockers, and antiarrhythmics in 53 (72%), 59 (80%), and 26 patients (35%), respectively. Details about antiarrhythmic agents are shown in Supplementary material online, *Table S2*. In addition, 30 patients (41%) underwent aetiology-driven therapy for VM, namely antiviral agents (*n* = 11), IVIG (*n* = 5), and IMT (*n* = 17). A total of 30 patients (41%) underwent ICD implant, whereas 24 (32%) received an implantable loop recorder. Further details about therapeutic choices are presented in the Supplementary material. Except for acute phase support and aetiology-driven strategies, therapy at discharge was comparable between polymorphic and monomorphic VA cases, as well as in VM vs. virus-negative myocarditis groups (see Supplementary material online, *Table S2*).

### Outcomes

The full list of short- and long-term MAE is presented in *Table 3*. At discharge, MAE had occurred in 18 patients (24%), including all-cause death (*n* = 3), severe heart failure requiring circulatory support (*n* = 10), advanced AVB (*n* = 3), and major VA (*n* = 4). Deaths were caused by the following: refractory heart failure in two patients with relevant comorbidities (systemic sclerosis, *n* = 1; chronic graft-vs.-host disease, *n* = 1), and intracranial haemorrhage in another patient with a malignant pheochromocytoma. After excluding *n* = 3 inhospital deaths and *n* = 3 patients undergoing catheter ablation of VA before discharge, the remaining 68 cases were eligible for long-term evaluation and completed the FU. By 24 months, MAE had occurred in 19 of them (28%) and included major VA in most cases (*n* = 15/19), who subsequently underwent either catheter ablation (*n* = 13) or modification of antiarrhythmic drugs (*n* = 2).

**Table 3:** Major adverse events at discharge and during follow-up

MAE by discharge	P-VA (*n* = 32)	M-VA (*n* = 42)	*P*	Total VM (*n* = 74)	Comparator VNM (*n* = 74)	*P*
All-cause death	3 (9)	0 (0)	0.077	3 (4)	1 (1)	0.620
Cardiac	2 (6)	0 (0)	0.184	2 (3)	1 (1)	1.000
Non-cardiac	1 (3)	0 (0)	0.432	1 (1)	0 (0)	1.000
Need for circulatory support	10 (31)	0 (0)	**<0.001**	10 (14)	3 (4)	0.078
Advanced AVB	3 (9)	0 (0)	0.077	3 (4)	2 (3)	1.000
Second-degree Mobitz 2	0 (0)	0 (0)	1.000	0 (0)	0 (0)	1.000
Third degree	3 (9)	0 (0)	0.077	3 (4)	2 (3)	1.000
Major VA	2 (6)	2 (5)	1.000	4 (5)	7 (9)	0.533
VF	1 (3)	0 (0)	0.432	1 (1)	1 (1)	1.000
Sustained VT	1 (3)	2 (5)	1.000	3 (4)	6 (8)	0.494
Appropriate ATP	0 (0)	0 (0)	1.000	0 (0)	0 (0)	1.000
Appropriate shock	0 (0)	0 (0)	1.000	0 (0)	0 (0)	1.000
Any MAE	16 (50)	2 (5)	**<0**.**001**	18 (24)	11 (15)	0.214

MAE by 24-month follow-up	P-VA (*n* = 26)	M-VA (*n* = 42)	*P*	Total VM (*n* = 68)	Comparator VNM (*n* = 70)	*P*
All-cause death	0 (0)	2 (5)	0.521	2 (3)	3 (4)	1.000
Cardiac	0 (0)	2 (5)	0.521	2 (3)	2 (3)	1.000
Non-cardiac	0 (0)	0 (0)	1.000	0 (0)	1 (1)	1.000
Rehospitalization for acute heart failure	0 (0)	2 (5)	0.521	2 (3)	4 (6)	0.681
Advanced AVB	0 (0)	1 (2)	1.000	1 (1)	2 (3)	1.000
Second-degree Mobitz 2	0 (0)	1 (2)	1.000	1 (1)	1 (1)	1.000
Third degree	0 (0)	0 (0)	1.000	0 (0)	1 (1)	1.000
Major VA	2 (8)	13 (31)	**0**.**034**	15 (22)	20 (29)	0.436
VF	0 (0)	0 (0)	1.000	0 (0)	0 (0)	1.000
Sustained VT	0 (0)	0 (0)	1.000	0 (0)	1 (1)	1.000
Appropriate ATP	1 (4)	4 (10)	0.642	5 (7)	3 (4)	0.490
Appropriate shock	1 (4)	9 (21)	0.076	10 (15)	16 (23)	0.278
Any MAE	2 (8)	17 (40)	**0**.**005**	19 (28)	25 (36)	0.364

Major adverse events in study groups and VA type subgroups are shown. Measures are count (%). Significant differences are enhanced in bold font. Additional events and outcomes are shown in Supplementary material online, *Table S3*.

ATP, antitachycardia pacing; AVB, atrioventricular block; MAE, major adverse events; VA, ventricular arrhythmias; M, monomorphic; P, polymorphic; VF, ventricular fibrillation; VM, viral myocarditis; VNM, virus-negative myocarditis; VT, ventricular tachycardia.

Overall, outcomes were comparable between cases with VM and matched controls with virus-negative myocarditis (*Table 3*; Supplementary material online, *Table S3*). Also, the use of aetiology-driven treatment was associated with a non-significant reduction of MAE occurrence (see Supplementary material online, *Table S4*).

### Risk stratification

**Figure 3:**
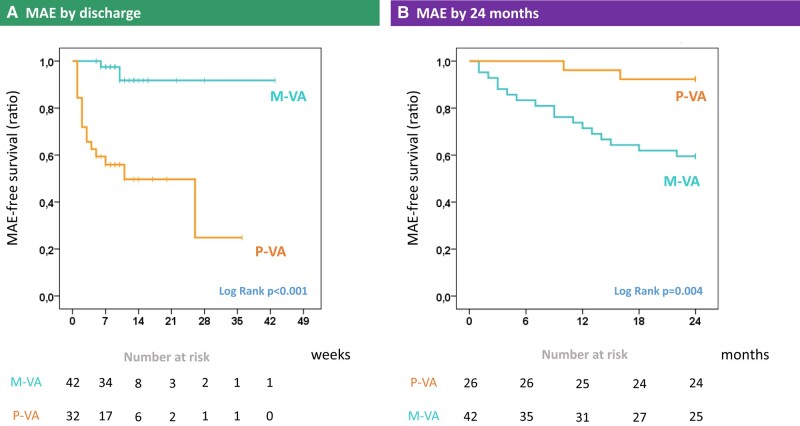
Kaplan–Meier curves in patients with polymorphic vs. monomorphic ventricular arrhythmias at presentation. Kaplan–Meier curves are shown for patients with viral myocarditis and early-phase polymorphic vs. monomorphic ventricular arrhythmias, for the endpoint of major adverse events (i.e. all-cause death, complicated heart failure, advanced atrioventricular blocks, or major ventricular arrhythmias). Results are shown both by hospital discharge on *n* = 74 patients (*A*, left) and by 24-month follow-up on *n* = 68 patients (*B*, right). Numbers at risk are reported under each chart. MAE, major adverse events; VA, ventricular arrhythmias; M, monomorphic; P, polymorphic.

**Figure 4:**
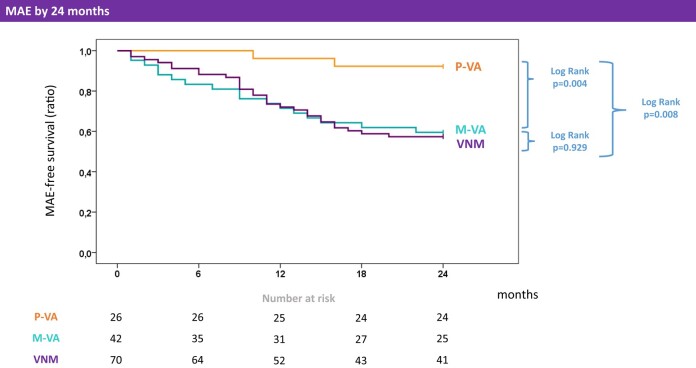
Outcomes in the study vs. comparator groups. Kaplan–Meier curves are shown for patients with viral myocarditis (*n* = 68) and early-phase polymorphic vs. monomorphic, as compared with control patients with virus-negative myocarditis (*n* = 70). Curves refer to the endpoint of major adverse events (i.e. all-cause death, complicated heart failure, advanced atrioventricular blocks, or major ventricular arrhythmias) by 24-month follow-up. Numbers at risk are reported under the chart. The significance of log rank test is shown for each couple of curves, as well as for all groups. MAE, major adverse events; VA, ventricular arrhythmias; M, monomorphic; P, polymorphic; VNM, virus-negative myocarditis.

At hospital discharge, MAE were more common in the polymorphic VA group compared to cases with monomorphic VA (16/32 vs. 2/42, *P* < 0.001). In particular, inhospital deaths were uniformly preceded by polymorphic VA (three of three cases). Conversely, the incidence of MAE during FU was higher in patients presenting with monomorphic VA (17/42 vs. 2/26, *P* = 0.005). The Kaplan–Meier curves for MAE in the monomorphic vs. polymorphic VA groups are shown in *Figure 3*. In contrast to the polymorphic VA group, the 24-month survival curve of patients presenting with monomorphic VA was comparable to the matched comparator cohort with virus-negative myocarditis (*Figure 4*; log rank *P* = 0.929).

Among virus-related variables, ongoing systemic infection and multiple genomes showed the strongest association with MAE at discharge (see Supplementary material online, *Table S5*). It is worth noting that the occurrence of MAE during FU was higher in patients with low-load parvovirus B19 compared with those with high viral load (49% vs. 19%, *P* = 0.048).

The complete results of risk stratification analysis are summarized in *Table 4*. While polymorphic and monomorphic VA were strongly associated with the occurrence of MAE at discharge or during FU, presentation with sustained VT/VF was found as an additional, independent predictor of adverse outcomes in both scenarios [respectively: hazard ratio (HR) 4.7, 95% CI 1.6–14.0, *P* = 0.005 and HR 6.3, 95% CI 2.3–17.6, *P* < 0.001]. No prognostic role was observed for antiarrhythmic therapy (*Table 4*). The Kaplan–Meier curves stratified by the severity of VA onset are shown in Supplementary material online, *Figure S2*.

**Table 4:** Risk stratification analysis

Parameter	Feature present	Feature absent	Univariable HR 95% CI	*P*	Multivariable Cox’s HR 95% CI	*P*
MAE by discharge						
Age > 40 years	12/49	6/25	1.0 (0.3–3.2)	1.000		
Male sex	10/49	8/25	0.5 (0.2–1.6)	0.390		
Systemic infection	16/34	2/40	16.9 (3.5–81.4)	**<0.001**	7.1 (1.2–32.6)	0.021
Multiple viral genomes	6/10	12/64	6.5 (1.6–26.7)	**0**.**011**		
Complicated^a^ presentation	13/33	5/41	4.7 (1.5–15.0)	**0**.**013**		
P-VA onset	16/32	2/42	20.0 (4.1–96.1)	**<0**.**001**	10.9 (2.2–55.2)	**0**.**004**
M-VA onset	2/42	16/32	0.05 (0.01–0.2)	**<0**.**001**		
Sustained VT/VF onset	11/20	7/54	8.2 (2.5–26.9)	**<0**.**001**	4.7 (1.6–14.0)	**0**.**005**
LVEF < 50%	9/22	9/52	3.3 (1.1–10.1)	**0**.**041**		
Septal LGE	10/32	8/42	1.9 (0.7–5.7)	0.279		
AAD use	13/53	5/21	1.0 (0.6–2.0)	0.896		
MAE by 24-month follow-up						
Age > 40 years	13/46	6/22	1.1 (0.3–3.3)	1.000		
Male sex	14/47	5/21	1.4 (0.4–4.4)	0.772		
Systemic infection	6/30	13/38	0.5 (0.2–1.5)	0.277		
Multiple viral genomes	2/9	17/59	0.7 (0.1–3.7)	1.000		
Complicated^a^ presentation	12/29	7/39	3.2 (1.1–9.7)	0.055	2.9 (0.6–28.1)	0.176
P-VA onset	2/26	17/42	0.1 (0.03–0.6)	**0**.**005**		
M-VA onset	17/42	2/26	8.2 (1.7–39.2)	**0**.**005**	16.1 (2.2–120)	**0**.**007**
Sustained VT/VF onset	10/16	9/52	8.0 (2.3–27.6)	**0**.**001**	6.3 (2.3–17.6)	**<0**.**001**
LVEF < 50%	5/20	14/48	0.8 (0.2–2.7)	1.000		
Septal LGE	10/28	9/40	1.9 (0.7–5.6)	0.278		
AAD use	14/49	5/19	1.1 (0.5–2.8)	0.618		

Results of univariable and multivariable analyses are shown in the cohort of patients with viral myocarditis (*n* = 75), referring to the endpoint of MAE (i.e. all-cause death, complicated heart failure, advanced atrioventricular blocks, or major VA) by discharge and by 24-month follow-up. Hazard ratios (HR) with 95% confidence intervals (CI) are shown. Significant differences are enhanced in bold font. Bonferroni correction for multiple testing was applied, resulting in a *P* < 0.017 as the modified threshold for statistical significance in the multivariable model.

AAD, antiarrhythmic drug; LGE, late gadolinium enhancement; LVEF, left ventricular ejection fraction; MAE, major adverse events; VA, ventricular arrhythmias; M, monomorphic; P, polymorphic; VF, ventricular fibrillation; VM, viral myocarditis; VNM, virus-negative myocarditis; VT, ventricular tachycardia.

^a^Complicated presentation included either acute heart failure, LVEF < 50%, or sustained VT/VF.^8^

## Discussion

### Main study findings

Our study was innovative in reporting clinical features and outcomes in a cohort of patients uniformly characterized by first diagnosis of VM and evidence of VA within 24 h of hospital admission. As compared with the prior publications from our group,^3–5^ this manuscript focused on EMB-proven viral rather than virus-negative aetiology, so that overlap was minimal and limited to the comparator group. Main study findings were as follows: (i) polymorphic VA were more common in patients with ongoing systemic infection (usually from cardiotropic viruses such as adenovirus), whereas monomorphic VA were more frequent in subjects with underlying chronic inflammatory cardiomyopathy (as commonly found in association with low-load parvovirus B19); (ii) while early-onset polymorphic VA were associated with adverse outcomes at hospital discharge, the occurrence of MAE during FU was higher in patients with monomorphic VA, who behaved similarly to virus-negative myocarditis. These data suggest that arrhythmic features of VA at presentation may constitute a valuable tool to guide clinical management, and improve risk prediction, in patients with VM.

### Ventricular arrhythmia characterization and myocardial inflammation

To the best of our knowledge, no prior studies attempted to systematically describe VA at presentation of VM. Both clinical and preclinical reports described early-onset VA in patients with acute myocarditis mediated by cardiotropic viruses.^18,19^ We hereby showed that cardiotropic viruses, as adenoviruses, were more commonly found in patients presenting with polymorphic VA compared to monomorphic VA (63% vs. 19%). As previously reported,^3,20^ these findings may actually point to different stages of myocardial inflammation. In particular, multiple clues within the polymorphic VA group, namely fever, higher C-reactive protein, lower LVEF, and higher proportion of EMB-proven necrosis (*Table 1*), are all indicators of an acute rather than chronic stage of myocarditis. In contrast, patients with monomorphic VA more commonly had the features of chronic inflammatory cardiomyopathy, as suggested by the higher prevalence of replacement fibrosis and myocyte hypertrophy on EMB, as well as by the lower proportion of abnormalities on T_2_-weighted sequences at CMR (*Table 1*). In this setting, the evidence of low-load parvovirus B19 genome in >50% of patients presenting with monomorphic VA (*Table 2*) supports the lack of a direct pathogenic role, as already demonstrated in patients with chronic dilated cardiomyopathy.^21^ On the other hand, it could be hypothesized that the cardiotropic and cytopathic nature of adenovirus^6,19^ may directly lead to polymorphic VA due to acute myocardial injury. Further evidence from preclinical studies is needed to elucidate the mechanisms leading to arrhythmias under specific viral infections. Meanwhile, the analysis of VA morphology may help to stage clinically suspected VM in patients with uncomplicated presentation and no strict indications to EMB,^6^ such as in cases with VEBs.^20^ Conversely, when the clinical presentation is complicated by acute heart failure or life-threatening VA,^2,8^ EMB remains the gold standard technique to clarify myocarditis aetiology and enable aetiology-driven therapeutic strategies.

### Risk stratification

One major finding of the present study was the association between baseline VA features and outcomes of VM. In fact, patients with polymorphic VA had greater incidence of MAE at discharge, whereas those with monomorphic VA had worse outcomes during FU. In keeping with prior reports, early-phase VA including VF may be life threatening and require circulatory support at third-level centres.^14,22^ Also in our experience, severe heart failure requiring haemodynamic assistance accounted for most of the short-term MAE. Instead, the incidence of major VA was low at discharge but higher in the long term. In this setting, recent data have shown that acute presentation with major VA was independently associated with major arrhythmic recurrences during FU.^23,24^ Consistently, while a watchful waiting strategy was advised in the past,^2,22^ the last ESC guidelines recommended ICD implant by discharge for the secondary prevention of sudden cardiac death in myocarditis.^1^ Even in VM, malignant VA may relapse independently of the degree of systolic dysfunction.^25^ Our study adds a piece of evidence in this setting, showing that an early protective strategy better applies to patients presenting with monomorphic VA (*Table 4*). In fact, in keeping with a scar-related reentry mechanism,^20^ our patients with monomorphic VA frequently showed either stable or progressive cardiomyopathic features (see Supplementary material online, *Table S3*) and outcomes comparable with virus-negative controls (*Figure 4*). Our preliminary findings deserve confirmation from large multicentre studies with a longer FU.

### Strengths and limitations

The main strengths of our study include the following: (i) restrictive inclusion criteria, resulting in the selection of 74 very well-characterized patients with evidence of VM out of a cohort of 796 patients with clinically suspected myocarditis; (ii) in the entire study population, the diagnosis of myocarditis was confirmed by EMB and frequently also by CMR^2,6^; (iii) uniform characterization of baseline VA by 12-lead ECG and availability of cardiac device telemonitoring in 73% of VM cases during FU^26^; (iv) 24-month FU available in all cases^4,5^; and (v) the prospective nature of the study.

Relevant study limitations include single-centre design, relative small sample size, and limited duration of FU. In turn, the specific focus of the study and subsequent exclusion of the ‘non-arrhythmic’ presentations of VM introduce a sampling bias for comparison. Restaging of myocarditis was not routinely performed during FU. In particular, EMB was not repeated to assess the persistence of myocardial viral genomes, as a well-known prognostic factor.^9^ No molecular tests were performed to test for virus transcriptional activity. Furthermore, our histological analysis did not include investigation of the conduction system as a potential arrhythmogenic factor. Although the analysis of VA features was found helpful for diagnostic and prognostic purposes, our findings are meant to complement rather than replace the key role of EMB and CMR in this setting.^2^ Finally, since the study design enabled patient-tailored choices, we could not provide convincing evidence about the effectiveness of any of the specific aetiology-driven therapies for VM. Dedicated randomized studies are needed to address that aim.

## Conclusions

In a population uniformly characterized by newly diagnosed VM and evidence of VA by 24 h of hospitalization, we showed that the type of arrhythmia at presentation is associated with distinct clinical scenarios and risk profiles. Specifically, while polymorphic VA were associated with ongoing systemic infection and greater incidence of adverse outcomes by discharge, presentation with monomorphic VA suggested chronic inflammatory cardiomyopathy and higher risk of MAE by 24 months, a condition largely comparable with virus-negative myocarditis. If confirmed by larger, prospective, multicentre studies, our findings may influence the management of arrhythmic myocarditis and improve risk.

## Supplementary Material

oead132_Supplementary_Data

## Data Availability

All data supporting the study results will be made available, upon reasonable request, by email correspondence with the corresponding author.

## Lead author biography

Giovanni Peretto, MD, FESC, is a Cardiac Electrophysiologist working at IRCCS San Raffaele Hospital and University, Milan, Italy. His main expertise is in the field of ventricular arrhythmias and myocarditis. Currently, Dr Peretto is the referral physician for the outpatient clinics of Arrhythmogenic and Inflammatory Cardiomyopathies at San Raffaele Hospital, as a part of a dedicated Multidisciplinary Disease Unit. As a passionate researcher, Dr Peretto is about to complete an International PhD Course in Molecular Medicine, to investigate the relationships between ventricular arrhythmias and myocardial inflammation, with the ultimate goal of identifying novel therapeutic targets by multidisciplinary, translational approach.
