# Supplementary material for: Clinical profiling and outcomes of viral myocarditis manifesting with ventricular arrhythmias
Source: Eur Heart J Open. 2023 Dec 5;3(6):oead132. doi: 10.1093/ehjopen/oead132 (PMC10733193; doi:10.1093/ehjopen/oead132)
Supplement: oead132_Supplementary_Data [file oead132_supplementary_data.docx]

**Clinical profiling and outcomes of viral myocarditis manifesting with ventricular arrhythmias**

**SUPPLEMENTARY MATERIAL**

Supplementary Methods (1), Tables (2), Figures (3), and References (4) are listed below.

**1. Supplementary Methods**

**Endomyocardial biopsy (EMB)**

Percutaneous right ventricular EMB was performed in all cases under fluoroscopic and echocardiographic guidance. Cardiac tissue (5 samples per patient) were analyzed at an external, referral center by experienced cardiac pathologists. In detail, 4 samples were immediately fixed in 10% buffered formalin at room temperature for light microscopy and immunohistochemistry, and 1 sample was stored in RNA later tubes at room temperature for viral polymerase chain reaction (PCR). PCR was performed on blood sample too, to evaluate for coexisting viremia.

EMB-proven myocarditis was defined, as recommended by the ESC (1), by histological, immunohistochemical and molecular criteria. In detail: a) histology: Dallas criteria, defined as histological evidence of inflammatory infiltrates within the myocardium associated with myocyte degeneration and necrosis of nonischemic origin; patients with no evidence of necrosis, but significant inflammatory infiltrates (“borderline” myocarditis) were included only when the clinical suspicion of myocarditis was confirmed by cardiac magnetic resonance (see below and main text) and in the presence of abnormal T-troponin (> 14 ng/L according to the local upper reference value); b) immunohistochemistry: presence of an abnormal inflammatory infiltrate, defined as follows: ≥ 14 leucocytes/mm2 including up to 4 monocytes/mm2 with the presence of CD 3 positive T-lymphocytes ≥ 7 cells/mm2; c) molecular biology to identify viral myocarditis. Based on the local epidemiology and local practice, in the absence of clinical or laboratory findings suggesting other infective etiologies, a standard panel of viruses was analyzed, including: Coxsackieviruses A and B, echoviruses, rhinoviruses, influenza A and B viruses, adenoviruses, parvovirus B19, cytomegalovirus, human herpes virus-6, Epstein-Barr virus, varicella-zoster virus, herpes simplex virus-1. In addition, the genome of the Severe Acute Respiratory Syndrome (SARS) coronavirus 2 has been systematically screened by the time of COVID-19 pandemic (since May 2020). In compliance with the position statement of the ESC (1), serological testing was applied only to limited viral etiologies, including human immunodeficiency virus (HIV) and hepatitis C virus (HCV).

**Classification of viral myocarditis**

Viral etiologies were classified according to multiple and updated criteria (2). In detail, the following classifications were applied, in compliance with updated evidence from virology and viral myocarditis (1,2).

| **Criterion** | **Group clustering** |
| --- | --- |
| By presence of systemic infection | with ongoing systemic infection (i.e. clinical presentation accompanied by any of the following: ongoing fever, viremia, and/or documentation of at least one extracardiac involvement) vs. without |
| By seasonality of infection | Spring vs. Summer vs. Autumn vs. Winter infection |
| By viral genome type | DNA virus vs. RNA virus |
| By selective tropism | cardiotropic (i.e. adenoviruses, enteroviruses) vs. vasculotropic (i.e. parvovirus B19) vs. lymphotropic (i.e. herpesviruses) vs. cardiotoxic (i.e. hepatitis C, HIV, influenza, SARS-CoV-2) |
| By number of viral genomes identified in cardiac tissue | single vs. multiple |
| By intramyocardial viral load | high-load (i.e. high viral copy number in cardiac tissue, rough equivalent of virulent infection) vs. low-load (i.e. low viral copy number in cardiac tissue, rough equivalent of bystander infections). Depending on the viral genome type, quantitative cutoffs were applied. In case of parvovirus B19 and human herpes virus 6, the cutoff of 500 viral DNA copies per microgram of cardiac DNA was applied as accepted (2). In the absence of universal standards (2), quantitative analysis was not performed for the remaining viruses. |

**Cardiac magnetic resonance (CMR)**

CMR was performed by a 1.5 T scanner (Achieva dStream; Philips Medical Systems, Eindhoven, The Netherlands) equipped with a 32-channel phased-array coil. Images were acquired according to the updated Society for Cardiac Magnetic Resonance recommendations (3) implemented with the evaluation of the updated Lake Louise criteria (LLC) (4). Myocardial edema was evaluated using black blood T2- short tau inversion recovery (STIR) images on two orthogonal planes. Late gadolinium enhancement (LGE) images were acquired 10 min after gadolinium injection using 2D T1 weighted segmented inversion-recovery gradient-echo sequences, and analyzed on two orthogonal planes. The correct inversion time was determined using the Look-Locker technique. In the most recent cases (n=44/74, 59%), native T1 and T2 mapping sequences were acquired in the short axis plane (base, mid-ventricle, apex). Modified Look-Locker inversion recovery sequences and gradient-(echo planar imaging) and spin-echo multi-echo sequences were used for T1 mapping and T2 mapping, respectively. Extracellular volume (ECV) was calculated as follows: ECV = (1 − hematocrit) × [ΔR1myocardium] / [ΔR1bloodpool], where ΔR1 is the difference in pre-contrast and post-contrast relaxation rates (1/T1) (4). All CMR images were analyzed by experienced readers blinded to baseline clinical assessment and patient outcomes, using a dedicated cardiac software (CVI42v.5.6.6, Circle Cardiovascular Imaging, Calgary, Canada).

**FDG-PET scan**

^18^F-Fluorodeoxy-glucose positron emission tomography (FDG-PET) did not contribute to the patient selection in the present study. Instead, it was used to allow follow-up restaging of myocarditis in patients with limitations to CMR (5). Standard preparation for FDG-PET consisted of a low-carbohydrate-high-fat diet for 12 hours, followed by a 12-hour fast, in order to achieve suppression of physiologic glucose myocardial metabolism (6). Thorax cardiac gated PET scan was acquired by PET/computed tomography (CT) scanner GE Healthcare (Xeleris Functional Imaging Workstation, GE; CardiolQ Physio, GE). Post-processing included both static and gated mode reconstructions, performed using a 3D algorithm integrating all the available models (TOF, PSF, Regularization). Parameters were optimized to obtain the best image quality, and 8-10 bins were used to perform gated mode reconstruction. Non-attenuation corrected images were evaluated in all patients in order to rule out the possibility of artifacts related to implantable cardioverter defibrillators (ICD). As previously described, positivity was deemed in the presence of focal or focal-on-diffuse pathological FDG uptake (5).

**VA definition and characterization**

As currently accepted (7), ventricular tachycardia (VT) was defined as > 3 consecutive complexes originating in the ventricles at a rate > 100 bpm: non sustained (NSVT), if spontaneously terminating within 30 s; or sustained, if lasting > 30 s or requiring termination due to hemodynamic compromise; ventricular fibrillation (VF) was defined as irregular electrical activity with ventricular rate > 300 bpm. During follow-up, major VA were defined as either sustained VT, VF, or appropriate ICD interventions (anti-tachycardia pacing or shock) for VT or VF.

Based on the Lown’s classification (8), only grade ≥ 2 ventricular ectopic beats (VEBs) were considered (> 1 VEB per minute, or > 30 VEBs per hour). Information analyzed about VEB (9) included: 1) number (absolute number in the first 24 h of telemonitoring); 2) 12-leads morphology: monomorphic (single VEB morphology, or one dominant representing ≥ 75% of total morphologies), or polymorphic (all of the remaining cases). For NSVT and VT, terms were adapted as follows (9): monomorphic (same beat-to-beat morphology within a single arrhythmic episode), or polymorphic (change in beat-to-beat morphology during the same arrhythmic episode).

The parameter k, indicating the coefficient of concordant judgement between cardiac electrophysiologists who independently analyzed VA morphology on ECG (theoretical range 0-1) was 1, indicating 100% of agreement (74 of 74 in cases with viral myocarditis; and 74 of 74 in controls with virus-negative myocarditis). Accordingly, the judgement of the third electrophysiologist was never required.

**Statistical analysis**

To identify 1:1 matched controls with virus-negative myocarditis, the propensity score method was used, accounting for the following baseline covariates.

| **Covariate** | **Variable type** | **Unit** |
| --- | --- | --- |
| Age at presentation | Continuous | years |
| Sex | Discrete, df=2 | -male  -female |
| Clinical presentation | Discrete, df=3 | -chest pain  -dyspnea  -syncope or palpitation |
| Baseline LVEF | Continuous | % |
| VA type | Discrete, df=2 | -VT/VF  -NSVT/VEBs |
| *df=degree of freedom; LVEF=left ventricular ejection fraction; NSVT=nonsustained ventricular tachycardia; VA=ventricular arrhythmias; VEBs=ventricular ectopic beats; VF=ventricular fibrillation; VT=ventricular tachycardia.* | | |

Two matched well-balanced cohorts were subsequently obtained and included in the final analysis for outcome measures. Covariate distributions before and after propensity score matching are shown in Table S1.

As for the multivariable analysis (Cox proportional hazard model), covariates were chosen based on the known clinical relevance.

| **Candidate predictor** | **Rationale** |
| --- | --- |
| Age > 40 y | Age influences outcome in myocarditis (10) |
| Male sex | Male sex has been associated with myocardial fibrosis in myocarditis (10) |
| Multiple viral genomes | Associated with adverse outcome in myocarditis patients (15) |
| Complicated presentation | Presentation complicated by heart failure or major arrhythmias are known to be associated with adverse outcomes (11) |
| Sustained VT/VF onset | Patients with sustained VT/VF onset are at an increased risk of recurrent malignant VA during follow-up (12). Consistently, the 2022 ESC guidelines recommend early implant of ICD in this setting (13) |
| LVEF < 50% | Associated with adverse outcome in myocarditis patients (11) |
| Septal involvement by LGE | Associated with adverse outcome in myocarditis (14) |

In addition to the above-defined covariates, VA features, as well as relevant viral-associated risk factors (Table S5), were implemented in the risk stratification model presented in Table 4.

**2. Supplementary Tables**

**Table S1**

**Propensity score matched variables**

|  | Before matching | | | After matching | | |
| --- | --- | --- | --- | --- | --- | --- |
|  | Total VM  (n=74) | Comparator VNM  (n=308) | p | Total VM  (n=74) | Comparator VNM  (n=74) | p |
| Age (y) | 47 ± 16 | 48 ± 15 | 0.612 | 47 ± 16 | 47 ± 15 | 1.000 |
| Male sex | 49 (66) | 222 (72) | 0.321 | 49 (66) | 48 (65) | 1.000 |
| Clinical presentation  Chest pain  Dyspnea  Syncope/palpitation | 27 (36)  21 (28)  26 (35) | 79 (26)  105 (34)  124 (40) | 0.082  0.409  0.431 | 27 (36)  21 (28)  26 (35) | 26 (35)  21 (28)  27 (36) | 1.000  1.000  1.000 |
| LVEF (%) | 51 ± 13 | 48 ± 13 | 0.076 | 51 ± 13 | 51 ± 13 | 1.000 |
| VA type at presentation  Sustained VT/VF  NSVT/VEB | 20 (27)  54 (73) | 109 (35)  199 (65) | 0.218  0.218 | 20 (27)  54 (73) | 21 (28)  53 (72) | 1.000  1.000 |

Comparison between matched variables in patients with VM (n=74) vs. controls with VNM is shown before and after the application of the propensity score method. Measures are mean ± standard deviation, or count (%).

*LVEF=left ventricular ejection fraction; NSVT=nonsustained ventricular tachycardia; VA=ventricular arrhythmias; VEBs=ventricular ectopic beats; VF=ventricular fibrillation; VM=viral myocarditis; VNM=virus-negative myocarditis; VT=ventricular tachycardia.*

**Table S2**

**Treatment strategies**

|  | P-VA  (n=32) | M-VA  (n=42) | p | Total VM  (n=74) | Comparator VNM  (n=74) | p |
| --- | --- | --- | --- | --- | --- | --- |
| Acute-phase treatment |  |  |  |  |  |  |
| Iv diuretics  Inotropes  MCS* | 16 (50)  10 (31)  7 (22) | 3 (7)  0 (0)  0 (0) | **<0.001**  **<0.001**  **0.002** | 19 (26)  10 (14)  7 (9) | 14 (19)  3 (4)  2 (3) | 0.284  0.078  0.166 |
| Etiology-driven therapy |  |  |  |  |  |  |
| Antiviral agents*  IVIG  IMT* | 5 (16)  5 (16)  6 (19) | 6 (14)  0 (0)  11 (26) | 1.000  **0.013**  0.580 | 11 (15)  5 (7)  17 (23) | 0 (0)  0 (0)  37 (50) | **<0.001**  0.058  **0.001** |
| Cardiac device implant |  |  |  |  |  |  |
| ICD (dual chamber or subcutaneous)  CRT-D  Loop recorders | 11 (34)  2 (6)  10 (31) | 14 (33)  3 (7)  14 (33) | 1.000  1.000  1.000 | 25 (34)  5 (7)  24 (32) | 27 (36)  6 (8)  26 (35) | 0.863  1.000  0.862 |
| Catheter ablation of VA |  |  |  |  |  |  |
| Sustained VT  NSVT  VEBs | 3 (9)  0 (0)  0 (0) | 0 (0)  0 (0)  0 (0) | 0.077  1.000  1.000 | 3 (4)  0 (0)  0 (0) | 3 (4)  0 (0)  1 (1) | 1.000  1.000  1.000 |
| Cardiological treatment at discharge |  |  |  |  |  |  |
| RAAS-inhibitors  Betablockers  Antiarrhythmic agents** | 24 (75)  26 (81)  16 (50) | 29 (69)  33 (79)  20 (48) | 0.613  1.000  1.000 | 53 (72)  59 (80)  26 (35) | 55 (74)  60 (81)  28 (38) | 0.853  1.000  0.865 |

Treatment strategies are shown in patients with VM and P-VA vs. M-VA, as well as for the whole cohort of VM (n=74) vs. controls with VNM. Measures are count (%). Significant differences are enhanced in bold font.

*Detail about the cohort of patients with VM (n=74) are presented here below:

-MCS (n=7): intra aortic balloon pump (n=1), left ventricular Impella (n=4), right ventricular Impella (n=1), veno-arterial extracorporeal membrane oxygenator (n=4), multiple devices (n=3).

-Antiviral agents (n=11): iv acyclovir (n=2), iv valgancyclovir (n=3), oral HAART (n=4), rivabirin and pegylated-interpheron alpha (n=1), zanamivir (n=1).

-IMT (n=17): iv steroids, 1 g for 3 days (n=4), 1 mg/kg tapered-down oral steroids plus 2 mg/kg titrated-up azathioprine for 12 months (n=6), 1 mg/kg tapered-down oral steroids plus 2-3 g titrated-up mophetil mycophenolate for 12 months (n=1), subcutaneous anakinra 100 mg for 3-6 months (n=5), oral hydroxychloroquine for 2 weeks (n=1).

**Antiarrhythmic agents in n=53 patients with VM included: amiodarone (n=27), sotalol (n=14), flecainide (n=8), propafenone (n=3), and hydroquinidine (n=1), with no significant differences between P- and M-VA subgroups.

*CRT-D=cardiac resynchronization therapy; ICD=implantable cardioverter defibrillator; IMT=immunomodulatory therapy; IVIG=intravenous immunoglobulins; MCS=mechanical circulatory support; NSVT=nonsustained ventricular tachycardia; RAAS=renin angiotensin aldosterone system; VA=ventricular arrhythmias (M-=monomorphic; P-=polymorphic); VEBs=ventricular ectopic beats; VF=ventricular fibrillation; VM=viral myocarditis; VNM=virus-negative myocarditis; VT=ventricular tachycardia.*

**Table S3**

**Additional outcomes by 24-month follow-up**

|  | P-VA  (n=26) | M-VA  (n=42) | p | Total VM  (n=68) | Comparator VNM  (n=70) | p |
| --- | --- | --- | --- | --- | --- | --- |
| 24-month symptoms |  |  |  |  |  |  |
| NYHA class | 1 (1-2) | 1 (1-2) | 1.000 | 1 (1-2) | 1 (1-2) | 1.000 |
| 24-month ECG |  |  |  |  |  |  |
| PQ (ms)  QRS duration (ms)  QTc (ms) | 171 ± 41  101 ± 23  412 ± 33 | 179 ± 39  105 ± 23  412 ± 32 | 0.423  0.488  1.000 | 175 ± 39  103 ± 23  412 ± 32 | 180 ± 40  105 ± 24  411 ± 33 | 0.459  0.618  0.857 |
| LBBB | 2 (8) | 4 (10) | 1.000 | 6 (9) | 6 (9) | 1.000 |
| 24-month arrhythmias |  |  |  |  |  |  |
| NSVT  Lown’s grade ≥ 2* VEBs  VEBs daily burden (10^3^)  AF | 6 (23)  4 (15)  0.7 (0.1-1.9)  0 (0) | 25 (60)  10 (24)  0.9 (0.2-2.5)  2 (5) | **0.006**  0.542  0.412  0.521 | 31 (46)  14 (21)  0.8 (0.2-2.0)  2 (3) | 36 (51)  17 (24)  0.9 (0.3-2.2)  3 (4) | 0.502  0.685  0.683  1.000 |
| 24-month blood exams |  |  |  |  |  |  |
| T-troponin (ng/L)  NTproBNP (pg/mL)  CRP (mg/L)  ESR (mm/h) | 6 (3-8)  122 (88-854)  2 (1-5)  6 (3-13) | 10 (3-16)  156 (93-1020)  2 (1-5)  7 (3-14) | 0.309  0.525  1.000  0.829 | 8 (3-13)  139 (90-943)  2 (1-5)  7 (3-14) | 10 (4-19)  164 (100-1186)  3 (1-6)  8 (3-16) | 0.267  0.491  0.871  0.762 |
| 24-month echocardiogram |  |  |  |  |  |  |
| LVEDVi (mL/m^2^)  LVEF (%)  LVEF< 50%  E/E’  TAPSE (mm)  Pericardial effusion | 63 ± 21  57 ± 11  6 (23)  7 ± 4  22 ± 3  0 (0) | 68 ± 23  53 ± 12  9 (21)  8 ± 4  22 ± 3  2 (5) | 0.371  0.173  1.000  0.320  1.000  0.521 | 66 ± 22  55 ± 13  15 (22)  8 ± 4  22 ± 3  2 (3) | 68 ± 22  53 ± 13  18 (26)  8 ± 4  22 ± 3  4 (6) | 0.594  1.000  0.692  1.000  1.000  0.681 |
| 24-month myocarditis restaging |  |  |  |  |  |  |
| Updated LLC+ on CMR  CD3+ > 7/mm2 on EMB  Viral genome  Abnormal FDG-PET | 2/13 (15)  0/1 (0)  0/1 (0)  0/4 (0) | 5/23 (22)  1/2 (50)  2/2 (100)  1/7 (14) | 1.000  1.000  0.333  1.000 | 7/26 (27)  1/3 (33)  2/3 (67)  1/11 (9) | 4/24 (17)  1/4 (25)  0/4 (0)  2/16 (13) | 0.501  1.000  0.143  1.000 |
| VT recurrences after follow-up ablation |  |  |  |  |  |  |
| Appropriate ICD shocks | 0/1 (0) | 1/12 (8) | 1.000 | 1/13 (8) | 1/15 (7) | 1.000 |

Additional outcomes by 24 months, meant to complete the content of Table 3, are shown for patients with VM and P-VA vs. M-VA, as well as for the whole cohort of VM (n=74) vs. controls with VNM. Measures are mean ± standard deviation, median (quartile 1-quartile 3), or count (%). Significant differences are enhanced in bold font.

*AF=atrial fibrillation; CD=cluster of differentiation; CMR=cardiac magnetic resonance; CRP=C-reactive protein; ECG=electrocardiogram; EMB=endomyocardial biopsy; ESR=erhythrocyte sedimentation rate; FDG-PET=18F-fluorodeoxyglucose positron emission tomography; ICD=implantable cardioverter defibrillator; LBBB=left bundle branch block; LLC=Lake Louise criteria; LVEDVi=left ventricular end-diastolic volume indexed; LVEF=left ventricular ejection fraction; NSVT=nonsustained ventricular tachycardia; TAPSE=tricuspid annular plane systolic excursion; VA=ventricular arrhythmias (M-=monomorphic; P-=polymorphic); VEBs=ventricular ectopic beats; VF=ventricular fibrillation; VM=viral myocarditis; VNM=virus-negative myocarditis; VT=ventricular tachycardia.*

**Table S4**

**Outcomes according to etiology-driven strategies**

| MAE by discharge | Total VM  (n=74) | Etiology-driven treatment  (n=30) | No etiology-driven treatment  (n=44) | p |
| --- | --- | --- | --- | --- |
| All-cause death  -After etiology-driven treatment | 3 (4)  - | 2 (7)  2 (7) | 1 (2)  1 (2) | 0.562  0.562 |
| Need for circulatory support  -After etiology-driven treatment | 10 (14)  - | 10 (33)  1 (3) | 0 (0)  0 (0) | **<0.001**  0.405 |
| Advanced AVB  -After etiology-driven treatment | 3 (4)  - | 2 (7)  0 (0) | 1 (2)  1 (2) | 0.562  1.000 |
| Major VA  -After etiology-driven treatment | 4 (5)  - | 2 (7)  0 (0) | 2 (4)  2 (4) | 1.000  0.511 |
| Any MAE  -After etiology-driven treatment | 18 (24)  - | 14 (47)  3 (10) | 4 (8)  4 (8) | **<0.001**  1.000 |
| MAE by 24-month follow-up | Total VM  (n=68) | Etiology-driven treatment  (n=27) | No etiology-driven treatment  (n=41) |  |
| All-cause death | 2 (3) | 1 (4) | 1 (2) | 1.000 |
| Rehospitalization for acute heart failure | 2 (3) | 0 (0) | 2 (5) | 0.514 |
| Advanced AVB | 1 (1) | 0 (0) | 1 (2) | 1.000 |
| Major VA | 15 (22) | 4 (15) | 11 (27) | 0.371 |
| Any MAE | 19 (28) | 5 (19) | 14 (34) | 0.181 |

Outcomes according to etiology-driven strategies are shown for the study group of patients VM (n=74). Measures are count (%). Significant differences are enhanced in bold font.

*AVB=atrioventricular block; MAE=major adverse events; VA=ventricular arrhythmias; VM=viral myocarditis.*

**Table S5**

**Virus-related prognostic factors**

|  | MAE by discharge | | | MAE by 24-month follow-up | | |
| --- | --- | --- | --- | --- | --- | --- |
|  | Feature present | Feature absent | p | Feature present | Feature absent | p |
| Systemic infection | 16/34 (47) | 2/40 (5) | **<0.001** | 6/30 (20) | 13/38 (34) | 0.277 |
| Spring  Summer  Autumn  Winter | 5/21 (24)  2/10 (20)  7/25 (28)  4/18 (22) | 13/53 (25)  16/64 (25)  11/49 (22)  14/56 (25) | 1.000  1.000  0.775  1.000 | 6/19 (32)  2/9 (22)  6/23 (26)  5/17 (29) | 13/49 (27)  17/59 (29)  13/45 (29)  14/51 (27) | 0.766  1.000  1.000  1.000 |
| DNA viruses RNA viruses | 15/64 (23)  4/12 (33) | 3/10 (30)  14/62 (23) | 0.698  0.470 | 17/59 (29)  3/11 (27) | 2/9 (22)  16/57 (28) | 1.000  1.000 |
| Cardiotropic viruses Vasculotropic viruses Lymphotropic viruses Cardiotoxic viruses | 5/14 (36)  7/42 (17)  7/19 (37)  3/9 (33) | 12/60 (22)  11/32 (34)  11/55 (20)  15/65 (23) | 0.289  0.103  0.213  0.679 | 3/13 (23)  11/40 (28)  5/16 (31)  0/9 (0) | 16/55 (29)  8/28 (29)  14/52 (27)  19/59 (32) | 1.000  1.000  0.757  0.053 |
| Multiple viral genomes | 6/10 (60) | 12/64 (19) | **0.011** | 2/9 (22) | 17/59 (29) | 1.000 |
| Parvovirus B19, all  Parvovirus B19, low load*  Parvovirus B19, high load* | 7/42 (17)  3/27 (11)  4/15 (27) | 11/32 (34)  15/47 (32)  14/59 (24) | 0.103  0.053  1.000 | 11/40 (28)  11/25 (44)  0/15 (0) | 8/28 (29)  8/43 (19)  19/53 (36) | 1.000  **0.048**  **0.007** |

Association between virus-related features and MAE both by discharge (18 events in n=74 patients) and by 24-month follow-up (19 events in n=68 patients) are shown. Measures are fractions (%). Significant differences are enhanced in bold font.

*DNA=deoxyribonucleic acid; MAE=major adverse events; RNA=ribonucleic acid.*

**3. Supplementary Figures**

**Figure S1**

**Viral genomes**

**
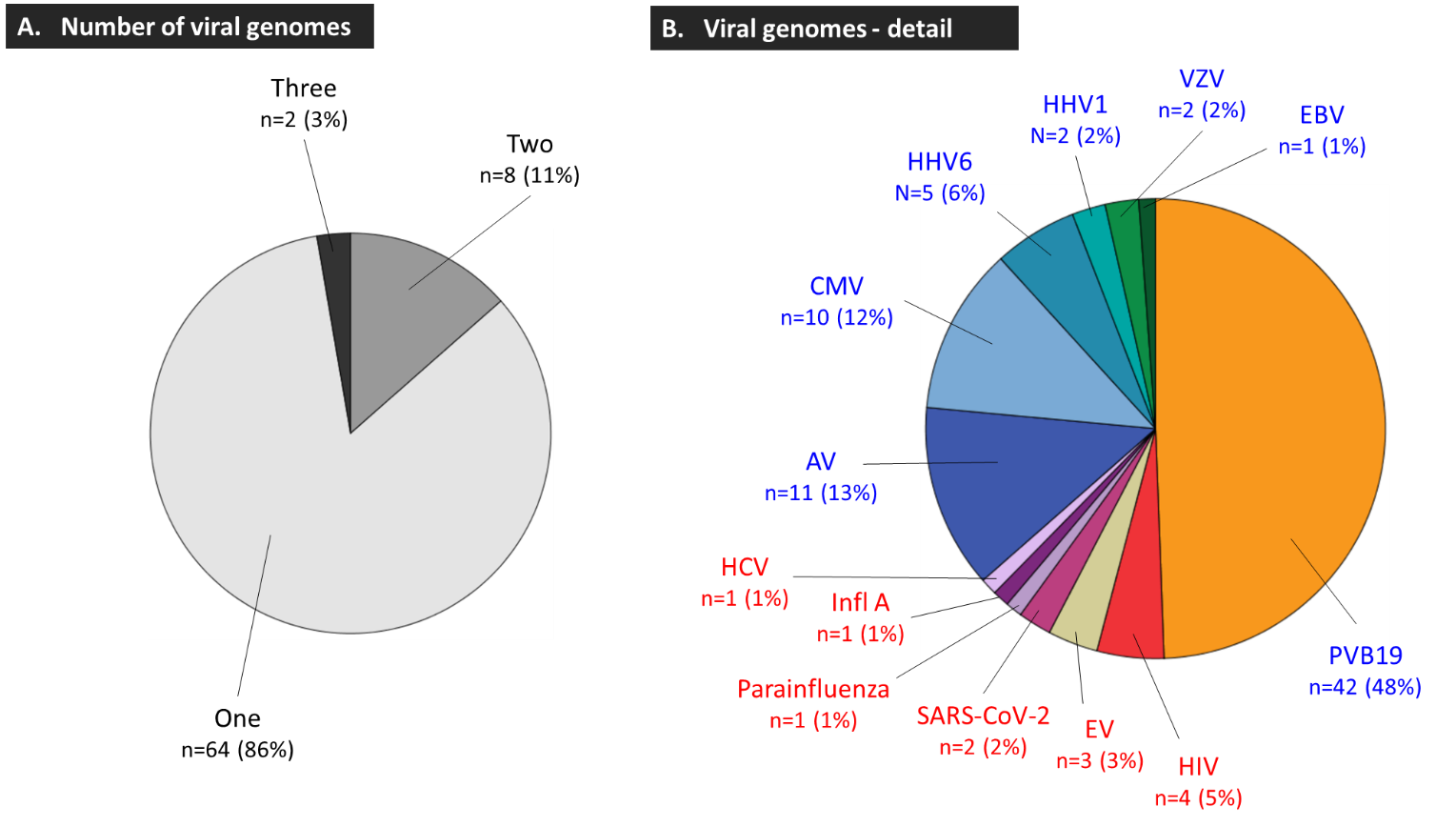
**

Detail about viral genomes associated with myocarditis (n=86 in n=74 patients) are shown, including number of viruses (panel A, left) and single genomes (panel B, right). In panel B, DNA genome viruses are labelled in blue font, while RNA genome viruses are labelled in red font. In panel B, percentages are referred to the total number of viral genomes rather than the total number of patients. For patients with multiple genomes, the observed combinations are hereby listed: AV+PVB19 (n=4), HIV+CMV (n=1); HCV+HHV6 (n=1), CMV+PVB19 (n=1), AV+CMV (n=1), AV+PVB19+CMV (n=1), AV+PVB19+HHV1 (n=1).

*AV=adenovirus; CMV=cytomegalovirus; EBV=Epstein-Barr virus; EV=enterovirus; HCV=hepatitis C virus; HHV=human herpes virus; HIV=human immunodeficiency virus; Infl A=influenza A virus; PVB19=parvovirus B19; SARS-CoV-2=severe acute respiratory syndrome coronavirus 2; VZV=varicella zoster virus.*

**Figure S2**

**Kaplan-Meier curves in patients with VT/VF vs. NSVT/VEB at presentation**


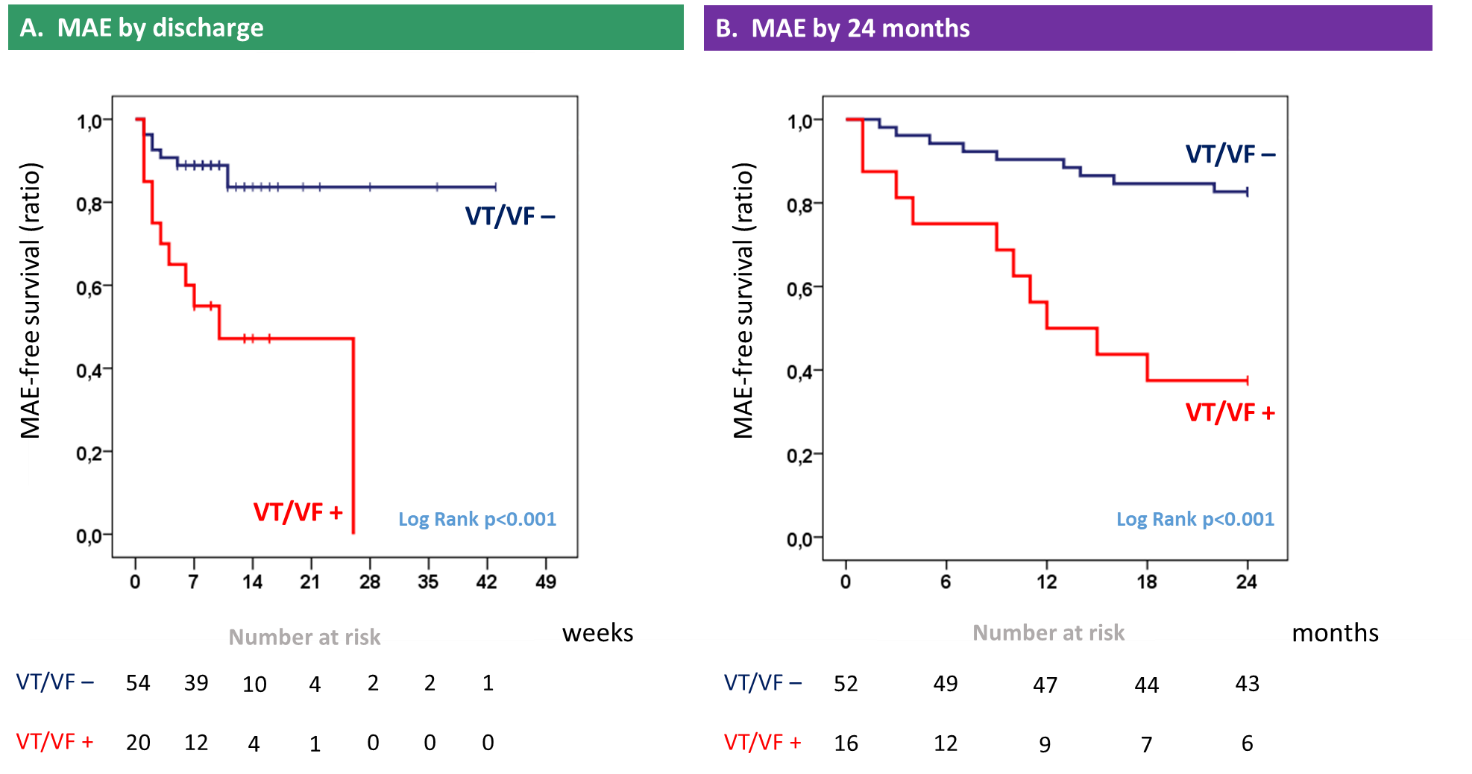


Kaplan-Meier curves are shown for patients with viral myocarditis and early-phase VT/VF (VT/VF+) vs. NSVT/VEBs (VT/VF-), for the endpoint of MAE (i.e. all-cause death, complicated heart failure, advanced atrioventricular blocks, or major VA). Results are shown both by hospital discharge on n=74 patients (panel A, left) and by 24-month follow-up on n=68 patients (panel B, right). Numbers at risk are reported under each chart.

*MAE=major adverse events; NSVT=nonsustained ventricular tachycardia; VEBs=ventricular ectopic beats; VF=ventricular fibrillation; VT=ventricular tachycardia.*

**4. Supplementary References**

1. Caforio AL, Pankuweit S, Arbustini E, et al. European Society of Cardiology Working Group on Myocardial and Pericardial Diseases. Current state of knowledge on aetiology, diagnosis, management, and therapy of myocarditis: a position statement of the European Society of Cardiology Working Group on Myocardial and Pericardial Diseases. Eur Heart J 2013;34:2636-2648.
2. Tschöpe C, Ammirati E, Bozkurt B, et al. Myocarditis and inflammatory cardiomyopathy: current evidence and future directions. Nat Rev Cardiol. 2021 Mar;18(3):169-193.
3. Kramer CM, Barkhausen J, Flamm SD, Kim RJ, Nagel E; Society for Cardiovascular Magnetic Resonance Board of Trustees Task Force on Standardized Protocols. Standardized cardiovascular magnetic resonance (CMR) protocols 2013 update. J Cardiovasc Magn Reson. 2013 Oct 8;15:91.
4. Ferreira VM, Schulz-Menger J, Holmvang G, at al. Cardiovascular Magnetic Resonance in Nonischemic Myocardial Inflammation: Expert Recommendations. J Am Coll Cardiol. 2018;72:3158-3176.
5. Peretto G, Busnardo E, Ferro P, et al. Clinical Applications of FDG-PET Scan in Arrhythmic Myocarditis. JACC Cardiovasc Imaging. 2022 Oct;15(10):1771-1780.
6. Manabe O, Yoshinaga K, Ohira H, et al. The effects of 18-h fasting with low-carbohydrate diet preparation on suppressed physiological myocardial (18)F-fluorodeoxyglucose (FDG) uptake and possible minimal effects of unfractionated heparin use in patients with suspected cardiac involvement sarcoidosis. J Nucl Cardiol. 2016;23:244-52.
7. Cronin EM, Bogun FM, Maury P, et al. 2019 HRS/EHRA/APHRS/LAHRS expert consensus statement on catheter ablation of ventricular arrhythmias. Europace 2019;21:1143-1144.
8. Oreto G, Satullo G, Luzza F, Donato A, Scimone IM, Cavalli A. Irregular ventricular tachycardia: a possible manifestation of longitudinal dissociation within the reentry pathway. Am Heart J 1992;124:1506-1511.
9. Peretto G, Sala S, Rizzo S, et al. Ventricular Arrhythmias in Myocarditis: Characterization and Relationships With Myocardial Inflammation. J Am Coll Cardiol. 2020;75:1046-1057.
10. Cocker MS, Abdel-Aty H, Strohm O, Friedrich MG. Age and gender effects on the extent of myocardial involvement in acute myocarditis: a cardiovascular magnetic resonance study. Heart 2009;95:1925-1930.
11. Ammirati E, Cipriani M, Moro C, et al. Registro Lombardo delle Miocarditi. Clinical presentation and outcome in a contemporary cohort of patients with acute myocarditis. Circulation 2018;138:1088-1099.
12. Peretto G, Sala S, Basso C, Della Bella P. Programmed ventricular stimulation in patients with active vs previous arrhythmic myocarditis. J Cardiovasc Electrophysiol. 2020 Mar;31(3):692-701.
13. Zeppenfeld K, Tfelt-Hansen J, de Riva M, et al. 2022 ESC Guidelines for the management of patients with ventricular arrhythmias and the prevention of sudden cardiac death. Eur Heart J. 2022 Oct 21;43(40):3997-4126.
14. Aquaro GD, Perfetti M, Camastra G, et al; Cardiac Magnetic Resonance Working Group of the Italian Society of Cardiology. Cardiac MR with late gadolinium enhancement in acute myocarditis with preserved systolic function: ITAMY Study. J Am Coll Cardiol 2017;70:1977-1987.
15. Kühl U, Pauschinger M, Seeberg B, et al. Viral persistence in the myocardium is associated with progressive cardiac dysfunction. Circulation. 2005 Sep 27;112(13):1965-70.
